# Postoperative radiotherapy may not be necessary for locally advanced head and neck squamous cell carcinoma: a case-match multicentre study

**DOI:** 10.1186/s12903-022-02288-x

**Published:** 2022-06-24

**Authors:** Zhen-Hu Ren, Jing-Shi Lei, Zhi-Min Yang, Sheng Zhang, Jian-Jun Yu, Han-Jiang Wu

**Affiliations:** 1 Department of Oral and Maxillofacial Surgery, Second Xiangya Hospital of Central South University, Changsha, Hunan China; 2 Department of Head and Neck Surgery, Hunan Cancer Hospital, Changsha, Hunan China; 3 Department of Oral and Maxillofacial-Head and Neck Oncology, Shanghai Jiao Tong University School of Medicine; College of Stomatology, Shanghai Jiao Tong University; National Center for Stomatology; National Clinical Research Center for Oral Diseases; Shanghai Key Laboratory of Stomatology, Shanghai, China; 4 Department of Oral Implantology, School and Hospital of Stomatology, Shanghai Engineering Research Center of Tooth Restoration and Regeneration, Tongji University, Shanghai, China

**Keywords:** Head and neck squamous cell carcinoma, Postoperative radiotherapy, Surgery alone, Overall survival, Disease-specific survival

## Abstract

**Background:**

Some head and neck cancer surgeons found that many patients with locally advanced head and neck squamous cell carcinoma (LA-HNSCC) without postoperative radiotherapy (PORT) also have a good prognosis. The purpose of this study was to determine the effect of PORT on survival in patients with LA-HNSCC.

**Methods:**

A case-match cohort analysis was performed at two institutions on patients with LA-HNSCC. Patients who received surgery alone were case-matched 1: 1 with patients treated by surgery plus PORT based on pT, pN, tumor subsite etc.

**Results:**

114 patients were matched into 57 pairs, with a median follow-up period of 40.2 months. No difference in overall survival (OS, HR 0.88; 95% CI 0.50–1.58; *P* = 0.79) or disease-specific survival (DFS, 0.86; 95% CI 0.50–1.50; *P* = 0.76) was observed with no PORT.

**Conclusions:**

PORT isn’t necessary for patients with LA-HNSCC who are treated for the first time as long as the head and neck cancer surgeon adhere to appropriate surgical concepts. The indications of PORT for patients with LA-HNSCC need to be further discussed.

## Background

Head and neck cancer is one of the most common malignancies, and squamous cell carcinoma (SCC) accounts for approximately 90% of all head and neck cancers [1]. Head and neck squamous cell carcinoma (HNSCC) arises in the oral cavity, oropharynx, larynx or hypopharynx and is the sixth leading cancer by incidence worldwide, with more than 600,000 cases diagnosed annually. Only 40%–50% of patients with HNSCC survive for 5 years [2]. Surgery, radiotherapy and chemotherapy are the mainstays of primary treatment in patients with HNSCC [3, 4]. However, despite the progress of multiple treatments and multidisciplinary treatments, the effect of treatment on HNSCC is not good, especially for locally advanced HNSCC (LA-HNSCC) [5, 6]. LA-HNSCC is cancer that has grown outside the origin organ but has not yet spread to distant parts of the body (Stage III/IV disease, pT3-4N0 or pT1-4N+). Most patients present with locoregionally advanced disease, and more than 50% have recurrence within 3 years [7]. At the same time, comprehensive treatment brings more complications and social and economic burden to patients. However, many experienced head and neck cancer surgeons have found that not all patients with LA-HNSCC, especially those with oral squamous cell carcinoma (OSCC) need adjuvant radiotherapy to achieve an ideal prognosis [8, 9]. Our previous study [10] (including some unpublished data) found that patients with locally advanced OSCC did not necessarily need postoperative radiotherapy (PORT) to achieve a good prognosis under the premise of high-quality surgical resection of the tumour.

Based on the above results and the clinical data of our unit, we propose two questions: (1) Is the benefit of PORT for head and neck squamous cell carcinoma as important as we expected and (2) Should the indication of PORT be further discussed? To answer these questions, we performed a case-match cohort analysis on patients with HNSCC at two institutions.

## Methods

### Eligibility criteria

Patients with newly diagnosed HNSCC between February 2010 and August 2016 were identified from two institutions. The retrospective study is approved by the ethics committee, and all participants provided written informed consent. Experimental protocols were approved by the appropriate institutional review committee (2019–222) and meet the guidelines of their responsible governmental agency. Patients are informed of all surgery-related and post-operative procedures and prognoses, and all patients are given their own choice of treatment. The including criteria were as follow: (1) HNSCC treated by primary surgery, including neck dissection; (2) cT3-4N0 or cT1-4N+; (3) at least 3-year follow-up. The exclusion criteria were: (1) previously treated HNSCC; (2) positive margin of tumour resection on histological examination; (3) distant metastasis; (4) incomplete follow-up data; (5) death within 1 month after surgery; (6) receiving PORT but not completing the radiotherapy plan and (7) age < 18 years.

### Surgical technique

**Fig. 1:**
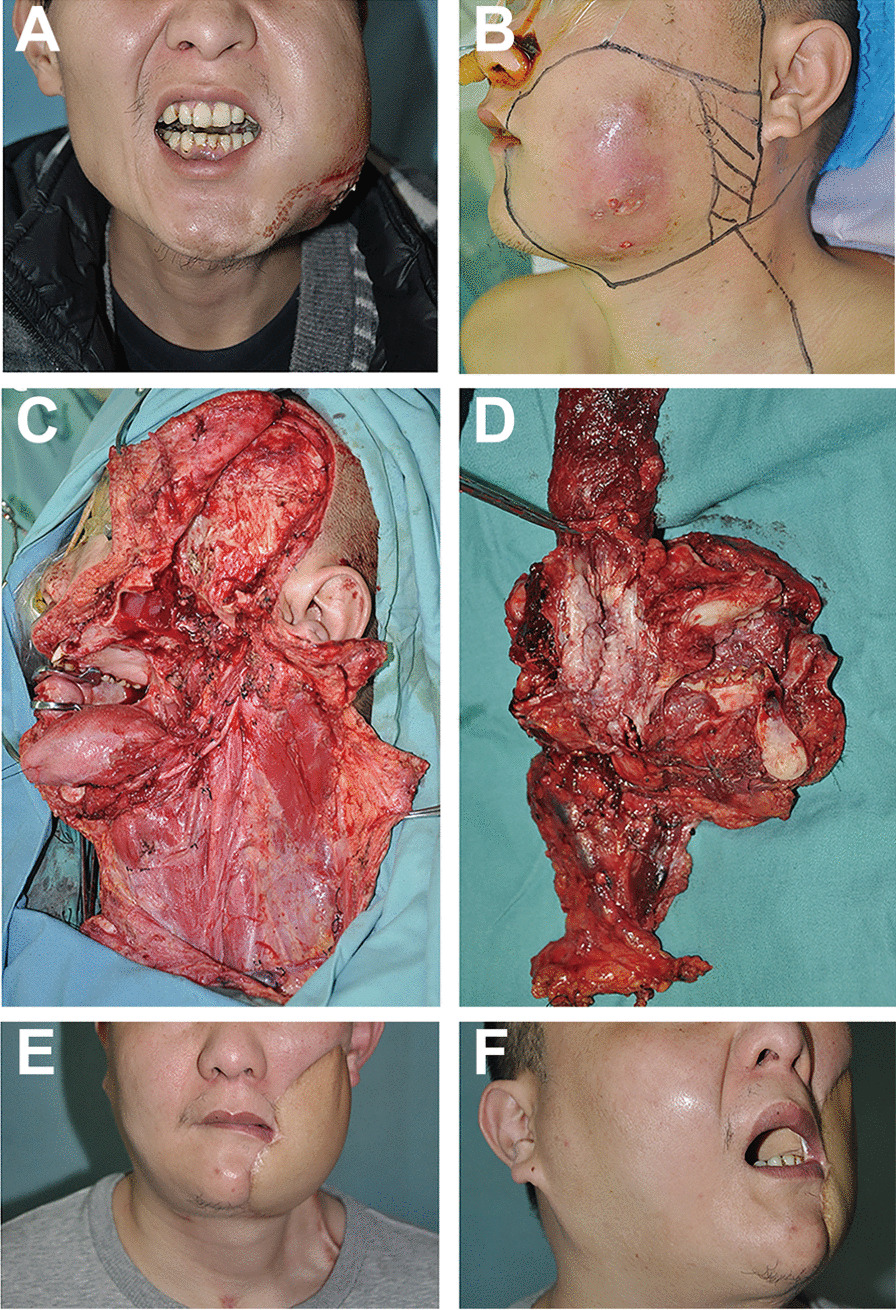
A typical case. **A**, **B** Preoperative photos and incision design; **C**, **D** Intraoperative photographs and tumor and cervical lymph node specimens; **E**, **F** 3 years after surgery

Our team has been committed to clinical research of the surgical treatment of HNSCC. Through long-term clinical studies, a large number of clinical cases were summarised. A series of improvements have been made to the surgical resection of HNSCC, which we call the anatomic unit (subunit) resection of HNSCC [10, 11]. The primary lesion excision was performed with anatomy unit resection surgery. All patients were performed radical resection of the primary lesion and neck dissection (suprascapulohyoid or full) with appropriate reconstruction (pedicle or free flap). The standard treatment of the surgery procedure is performed unified by two professional surgeons. All patients received *en bloc* excision with primary tumour excision combined with neck dissection. During the surgery, frozen sections were performed to confirm adequate margins. Figure 1 shows a typical case.

### Postoperative radiotherapy

Radiotherapy was started 4–6 weeks after surgery. A dose of 1.8–2 Gy per day, 5 days per week, for 6 weeks (54–60 Gy in total) was used as standard conformal or intensity-modulated radiotherapy. A total radiation dose of 66 Gy was recommended in patients with high-risk features. A small number of high-risk patients received concurrent chemotherapy with PORT.

The irradiation field includes the scope of the primary tumor and subclinical (including postpharyngeal lymph nodes) before surgery. All cases require postoperative CT/MRI scans for postoperative radiotherapy reference. Clinical target volume (CTV) and planning target volume (PTV) are defined as follows:

CTV: (1) Primary tumor before surgery; (2) In patients with positive lymph nodes, the area of the affected lymph nodes found on clinical and imaging studies (CT or MRI scans). (3) In the primary area and neck, potentially subclinical affected areas in the microscope. (4) CTV1 (High-risk): a area with visible lesions (i.e., tumor bed/operating bed) found by clinical or radiological methods before surgery, and/or pathological examination revealed positive borders/lymph node invasion + extracapsular spread/no extracapsular spread multiple lymph node invasion. (5) CTV2 (low risk): is considered a potential subclinical lesion but not in a high-risk area.

PTV: (1) PTV1 needs to cover CTV1; (2) PTV2 needs to cover CTV2.

### Grouping and pairing

A case-match cohort analysis (1:1) was performed between patients who were treated with surgery plus PORT and patients treated with surgery alone, eliminating patients who received chemotherapy after surgery. The investigator was blinded to the outcome during the matching. The included criteria is based on cT category. However, to make the statistical analysis more accurate, we used pT category in patient grouping. The hierarchy of matching was as follows: (1) pT category; (2) pN category; (3) tumour subsite; (4) age of the patient; (5) sex of the patient and (6) tumour differentiation.

### Follow-up and outcomes

After completion of operation (surgery-alone) or radiotherapy (surgery plus PORT), patients were monitored every month during the first year, every 3 months during the second year, every 6 months during the third year, and once per year thereafter until death or data censoring. At each follow-up visit, the patients underwent a standard postoperative assessment performed upon hospital admission. The assessment included the following: head and neck/abdomen ultrasound examination (every visit), chest X-rays (every 6 months), head and neck computed tomography (CT) (every 6 months), head and neck magnetic resonance imaging (MRI) (every 6 months) and positron emission tomography (PET)-CT (if required). If the patients did not return, we contacted the patient or his/her family to inquire about the patient’s condition. Overall survival (OS) was calculated from the date of operation to the date of death. Disease-free survival (DFS) were calculated from the date of operation to recurrence or death resulting from any cause.

### Statistical considerations

The primary end point was OS and the secondary end point was DFS. For descriptive analysis, categorical data were expressed as number and percentage. The survival analysis was conducted using the Kaplan–Meier method and log-rank test. Hazard ratios (HRs) were calculated using the Cox proportional hazards model. All hypothesis-generating tests were two sided, at a significance level of.05. Statistical analysis was performed using SPSS 22.0 (SPSS, Inc., Chicago, IL, USA) and GraphPad Prism 6.

## Results

**Fig. 2:**
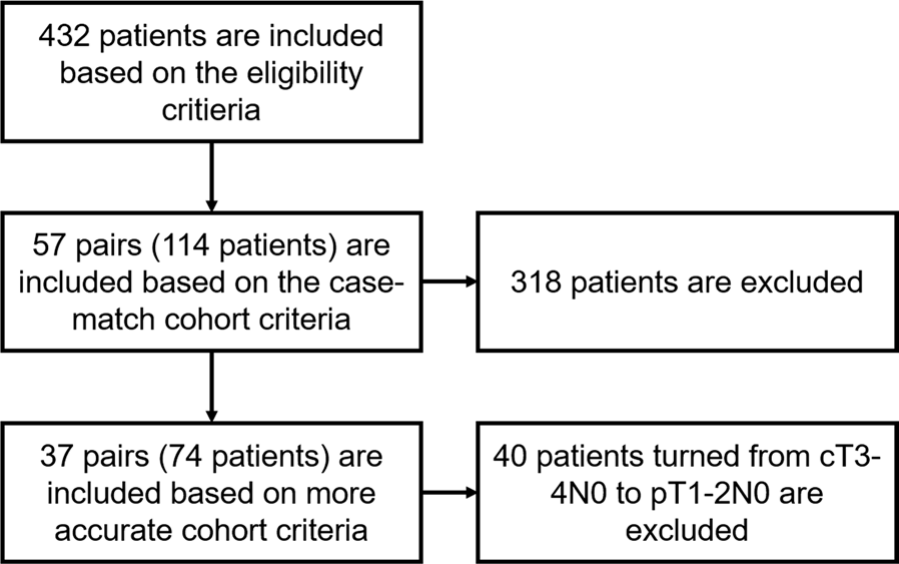
The illustration of the selection criteria and the relevant reasons for exclusion

A total of 432 patients were eligible for this study. After performing the case-match-designed matching, only 57 pairs (114 patients) were included. The quality of the matching was excellent, as summarised in Table 1. *P* values for chi-square tests of homogeneity were greater than 0.4 for all matches and for all variables except groups. 57 matched pairs of patients (110 men and 4 women) with a mean age of 51 ± 9.8 years were identified. The median follow-up period was 40.2 months (range 3–101 months). All patients had LA-HNSCC. Of the 57 patients who received adjuvant treatment, surgery with radiotherapy were 44 patients, other 13 had surgery with chemoradiotherapy. In statistical analysis, some included patients turned from cT3-4N0 to pT1-2N0. In order to be more accurate, we eliminated pT1-2N0 patients. The characteristics were summarised in Table 2. 37 pairs of patients (71 men and 3 women) with a mean age of 48 ± 9.9 years were remained. Of the 37 patients who received adjuvant treatment, surgery with radiotherapy were 28 patients, other 9 had surgery with chemoradiotherapy. The study selection criteria, including the relevant reasons for exclusion, are illustrated in Fig. 2.

**Table 1:** Clinical and pathologic characteristics

	Surgery only (n = 57), n(%)	Surgery + adjuvant (n = 57), n(%)	*P*
Age	51.4 ± 9.7	50.3 ± 9.1	0.876
Gender			0.999
Male	55	55	
Female	2	2	
Subsite			0.891
Tongue	32	30	
Buccal	16	17	
Floor of mouth	4	5	
Gingiva	2	2	
Oropharynx	2	3	
Lip	1	0	
pT			0.732
1	10	9	
2	42	43	
3	4	4	
4	1	1	
pN			0.999
0	22	22	
1	10	10	
2	25	25	
Pathological differentiation			0.999
Well	22	22	
Moderately	35	35	
Poorly	0	0	
Perineural invasion			0.574
Positive	27	30	
Negative	30	27	
Treatment			–
Surgery only	54	–	
Surgery + chemotherapy	3	–	
Surgery + radiotherapy	–	44	
Surtery + chemoradiotherapy	–	13	
Smoking			0.536
Yes	42	46	
No	15	11	
Alcohol			0.452
Yes	33	30	
No	24	27

**Table 2:** Clinical and pathologic characteristics (after elimination)

	Surgery only (n = 37), n(%)	Surgery + adjuvant (n = 37), n(%)	*P*
Age	50.6 ± 11.3	50.3 ± 8.4	0.898
Gender			0.556
Male	36	35	
Female	1	2	
Subsite			0.233
Tongue	21	22	
Buccal	3	3	
Floor of mouth	10	11	
Gingiva	1	1	
Oropharynx	1	0	
Lip	1	0	
pT			0.983
1	9	9	
2	23	24	
3	4	3	
4	1	1	
pN			0.999
0	2	2	
1	10	10	
2	25	25	
Pathological differentiation			0.812
Well	15	14	
Moderately	22	23	
Poorly	0	0	
Perineural invasion			0.485
Positive	17	20	
Negative	20	17	
Treatment			–
Surgery only	35	–	
Surgery + chemotherapy	2	–	
Surgery + radiotherapy	–	28	
Surgery + chemoradiotherapy	–	9	
Smoking			0.601
Yes	26	28	
No	11	9	
Alcohol			0.352
Yes	21	17	
No	16	20

**Fig. 3:**
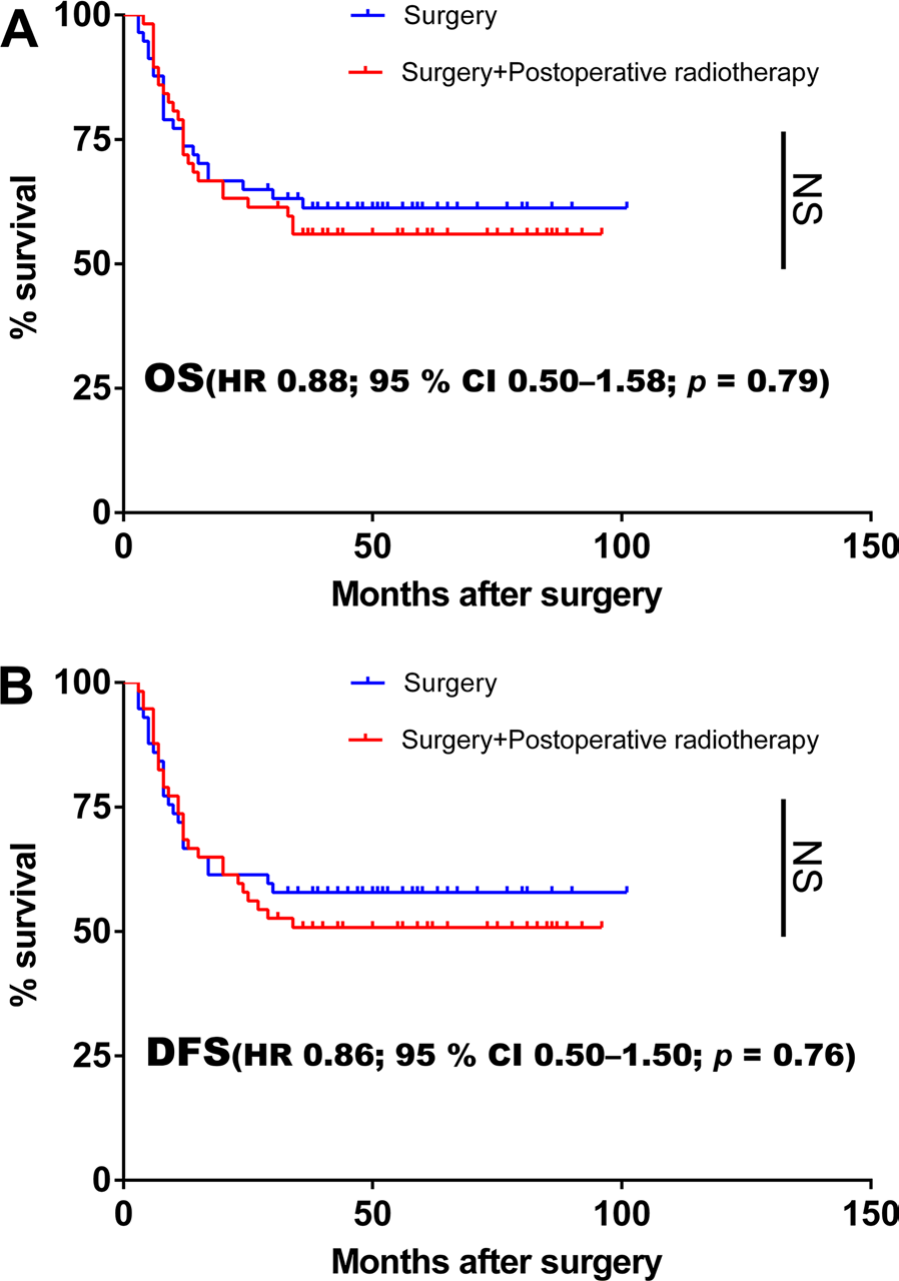
**A** No significant difference was found in OS between the two arms (HR 0.88; 95% CI 0.50–1.58; *P* = 0.79). **B** Similarly, there was no significant difference in DFS between the two groups (HR 0.86; 95% CI 0.50–1.50; *P* = 0.76)

**Fig. 4:**
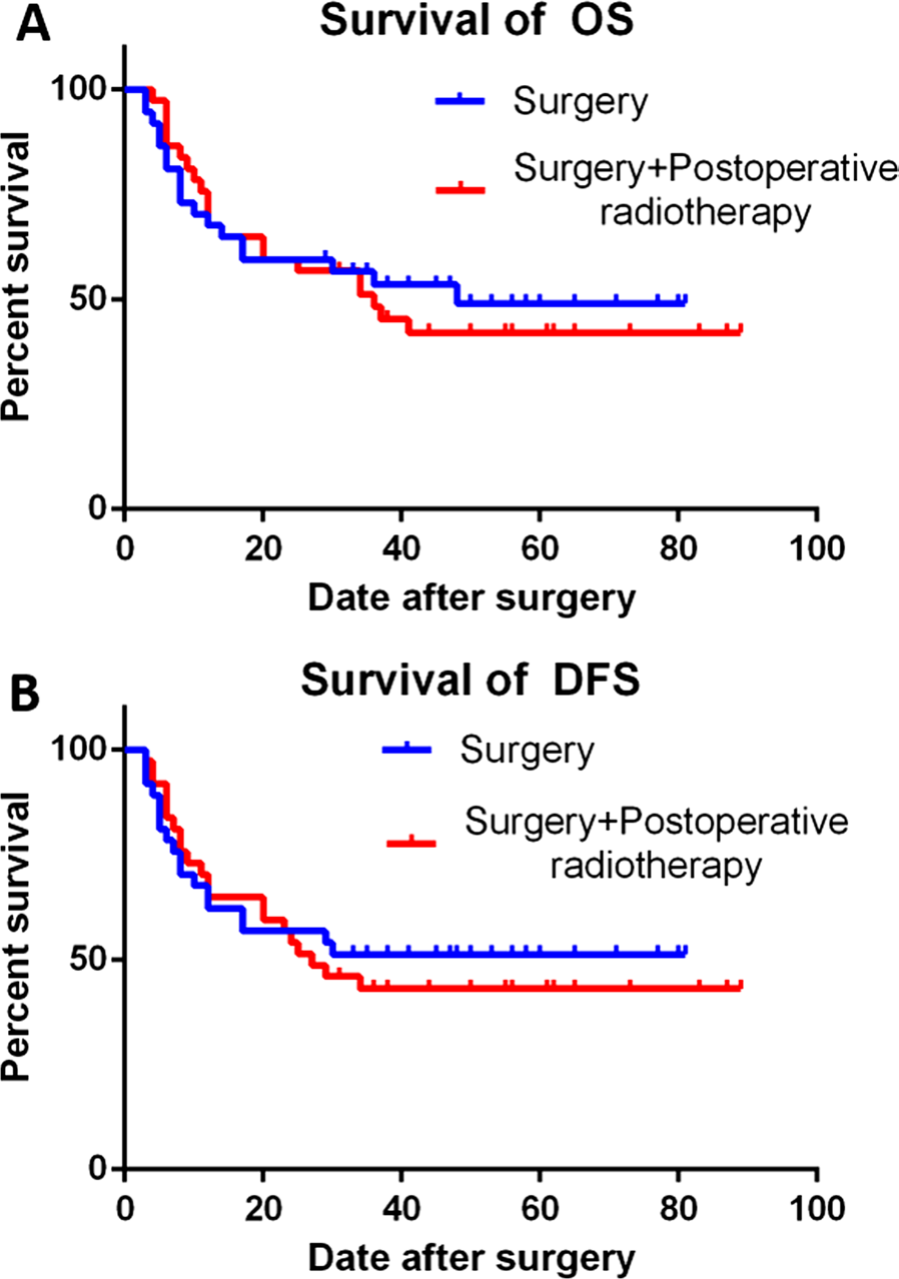
**A** No significant difference was found in OS between the two arms (HR 0.90; 95% CI 0.48–1.69). **B** Similarly, there was no significant difference in DFS between the two groups (HR 0.88; 95% CI 0.47–1.65)

The 3-year estimated OS rate was 57.9% for those treated with surgery alone and 54.4% for those receiving surgery plus PORT. There was no significant difference in OS between the two arms (HR 0.88; 95% CI 0.50–1.58; *P* = 0.79; Fig. 3A). Similarly, there was no significant difference in DFS between the two groups (HR 0.86; 95% CI 0.50–1.50; *P* = 0.76; Fig. 3B). The 3-year DFS was 52.6% and 49.1% in the surgery-alone group and surgery plus PORT, respectively. Locoregional recurrence occurred in 24 cases in the surgery-alone group and 26 in surgery plus PORT group. There was no statistically significant difference between the two groups. The surgery-alone group had three patients with distant metastasis, and all three patients had pulmonary metastasis, one of whom had multiple metastasis, including adrenal gland and pelvis. The surgery plus PORT group had three distant metastasis: two to the lungs and one to the ribs. After the elimination, the result was the same. No differences in OS (HR 0.90; 95% CI 0.48–1.68; Fig. 4A) or DFS (HR 0.88; 95% CI 0.47–1.65; Fig. 4B) were observed.

Uni- and multi-variable analyses are shown in Table 3. In univariate analysis, only subsite and lymph node metastasis were associated with an increased risk of OS (RR = 1.331; 95% CI 1.063–1.667, 1.638; 95% CI 1.164–2.305, respectively), DFS (RR = 1.345; 95% CI 1.084–1.669, 1.726; 95% CI 1.241–2.400, respectively). Adjuvant chemotherapy resulted in a significant improvement in DFS (RR 0.483; 95% CI 0.248–0.943). In multivariable analysis, after control for age, smoking and alcohol status, no difference in OS (RR 1.184; 95% CI 0.649–2.162) or DFS (RR 1.169; 95%CI 0.659–2.073) was observed with the addition of PORT. Subsite was associated with an increased risk of OS (RR 1.457; 95% CI 1.122–1.890), and DFS (RR 1.423; 95% CI 1.104–1.834). Lymph node metastasis was associated with an increased risk of OS (RR 1.628; 95% CI 1.143–2.317), and DFS (RR 1.725; 95% CI 1.227–2.424). After the elimination, Subsite and lymph node metastasis were associated with an increased risk of OS and DFS in the remaining 37 pairs.

**Table 3:** Uni- and multivariable analysis of outcomes

	OS RR (95% CI)	*P*	DFS RR (95% CI)	*P*
Age	0.989 (0.961–1.019)	0.472	1.000 (0.974–1.027)	0.993
Gender	2.013 (0.278–14.603)	0.489	2.302 (0.318–16.664)	0.409
Smoking	0.646 (0.345–1.207)	0.171	0.784 (0.425–1.446)	0.435
Alcohol	0.828 (0.468–1.468)	0.296	0.878 (0.509–1.512)	0.638
Subsite	1.331 (1.063–1.667)	**0.013**	1.345 (1.084–1.669)	0.007
T stage	1.129 (0.674–1.892)	0.645	1.036 (0.632–1.701)	0.887
Lymph node	1.638 (1.164–2.305)	**0.005**	1.726 (1.241–2.400)	0.001
Pathological grade	1.420 (0.769–2.623)	0.263	1.700 (0.933–3.100)	0.083
Postoperation radiotherapy	1.129 (0.636–2.002)	0.679	1.158 (0.671–1.997)	0.599
Adjuvant chemotherapy	0.587 (0.283–1.215)	0.130	0.483 (0.248–0.943)	0.033
Multivariable				
Subsite	1.457 (1.122–1.890)	0.005	1.423 (1.104–1.834)	0.006
Lymph node	1.628 (1.143–2.317)	0.007	1.725 (1.227–2.424)	0.002
Pathological grade	1.383 (0.734–2.605)	0.316	1.590 (0.851–2.970)	0.146
Postoperation radiotherapy	1.184 (0.649–2.162)	0.582	1.169 (0.659–2.073)	0.594
Adjuvant chemotherapy	0.647 (0.298–1.406)	0.272	0.579 (0.283–1.186)	0.135

Based on the case-match cohort analysis of 57 pairs, 37 patients relapsed within 1 year after surgery, including 19 patients in the surgery-alone group and 18 patients in the surgery plus PORT group. A total of 46 patients (including the 37 patients who relapsed in 1 year) relapsed 2 years after surgery (22 in the surgery-alone group and 24 in the surgery plus PORT group). A total of 57 patients (27 in the surgery-alone group and 30 in the surgery plus PORT group) relapsed within 3 years after surgery. The mean recurrence time of patients was 33.7 months, the median recurrence time was 30 months, and the recurrence cases within 1 year accounted for 32.5% (37/114) of all the recurrence cases. After eliminating pT1-2N0 patients including 37 pairs, 27 patients relapsed within 1 year after surgery, including 14 patients in the surgery-alone group and 13 patients in the surgery plus PORT group. 33 patients (including the 27 patients who relapsed in 1 year) relapsed 2 years after surgery (16 in the surgery-alone group and 17 in the surgery plus PORT group). A total of 44 patients (21 in the surgery-alone group and 23 in the surgery plus PORT group) relapsed within 3 years after surgery. The mean recurrence time of patients was 33.2 months, the median recurrence time was 29 months, and the recurrence cases within 1 year accounted for 36.5% (27/74) of all the recurrence cases.

## Discussion

This paper is a multi-institutional matched study of surgery-alone compared with surgery plus PORT in patients with LA-HNSCC. Our results showed that PORT did not improve OS or DFS in patients with LA-HNSCC when compared with surgery alone. The results are strengthened by the strict matching criteria. Two large head and neck cancer centres were utilised to recruit patients. Two institutions have adopted very similar treatment paradigms in their approaches to LA-HNSCC with a preference for withholding PORT wherever possible.

PORT has been included in the treatment guidelines for LA-HNSCC for about 50 years [12]. The results of our previous clinical study showed that patients with LA-HNSCC could obtain a good prognosis without PORT [10, 11, 13]. We designed this multicentre case-matching study to validate the conclusions of our previous study. The advantage of the case-matching study is that the factors and conditions affecting the experiment can be controlled in advance to make it as balanced as possible, reduce errors, and at the same time reduce the individual differences of the experimental subjects (patients). In order to eliminate the prescription bias associated with cohort studies comparing outcomes for patients receiving surgery alone with those treated by surgery plus PORT, we applied rigid matching criteria. Although minimised, the prescription bias cannot be completely eliminated. Medical comorbidity seriously affects the survival of patients, and because of the limited amount of data in our database, we did not use this factor as a match. That is unavoidable in all types of clinical studies, including RCTs. Meanwhile, the surgical techniques used in this study are similar to those of some scholars [14, 15]. The basic concept for the anatomic unit (subunit) resection is removal of the entire anatomical subunit in which tumour is contained rather than removing tumour with a 1–2 cm histopathological margin. The results of this clinical study showed that PORT did not significantly improve the OS rate or tumour-free survival rate of patients with LA-HNSCC. Multivariate analysis showed that only lymph node metastasis and tumour site were independent factors affecting survival.

In contrast with our study, many other trials [16–18] show that PORT shows an improvement in the outcome of patients with LA-HNSCC. The study by Yanamoto et al. [16] showed that after the propensity score analysis, PORT/concurrent chemoradiotherapy significantly improved OS (HR 0.554; 95% CI 0.38–0.80; *P* = 0.001) and DSS (HR 0.641; 95% CI 0.43–0.96; *P* = 0.030) compared to surgery only. The finding of Wang et al. [17] suggested that patients undergoing surgery had a 5-year OS of 27% compared with 66% for patients undergoing surgery plus adjuvant radiotherapy (*P* = 0.003); patients undergoing surgery had a 5-year DFS of 34% compared with 74% for patients undergoing surgery plus adjuvant radiotherapy (*P* = 0.001). However, compared with our study, these studies had some differences. Firstly, by the prescription bias associated with an unmatched comparative study of this nature, these above data are confounded; case-matching study can minimise the error in this aspect. Secondly, the concept of surgery in our study is different from that in the above studies. Another two case-matching studies [19, 20] came to the similar conclusion as our study: with the addition of adjuvant radiation (or radiation and chemotherapy), no difference in OS or DSS was observed. Another possible reason is that only five of the 114 patients in this study were oropharyngeal SCC and the rest were OSCC.

About the local toxicity associated with PORT on health-related quality of life, the negative impact is well documented [21, 22]. During and after treatment, overall health-related quality of life declines, and it will be at least 1 year before the baseline levels are recovered. However, physical function related to saliva, swallowing and chewing remain persistently low [23]. For conventional radiotherapy compared to intensity-modulated radiotherapy, this effect is worse [24].

## Conclusions

Based on the results of our clinical trial, we conclude that PORT does not improve the OS of patients with LA-HNSCC, but may benefit the DFS of patients. Therefore, we believe that PORT is not necessary for patients with LA-HNSCC who are treated for the first time as long as the head and neck cancer surgeon adheres to appropriate surgical concepts (specifically the anatomic unit (subunit) resection). The evidence for this conclusion is not strong enough. Furthermore, the prognoses of the PORT for different surgical approaches need to be confirmed by other high-quality clinical studies.

## Data Availability

The datasets generated and analysed during the current study are not publicly available due to the requests of hospitals but are available from the corresponding author on reasonable request.

## Abbreviations

LA-HNSCC: Locally advanced head and neck squamous cell carcinoma
HNSCC: Head and neck squamous cell carcinoma
PORT: Postoperative radiotherapy
OS: Overall survival
DFS: Disease-specific survival
RCTs: Randomized controlled trials
SCC: Squamous cell carcinoma
OSCC: Oral squamous cell carcinoma
